# High Quality Genomic Copy Number Data from Archival Formalin-Fixed Paraffin-Embedded Leiomyosarcoma: Optimisation of Universal Linkage System Labelling

**DOI:** 10.1371/journal.pone.0050415

**Published:** 2012-11-29

**Authors:** Abdulazeez Salawu, Aliya Ul-Hassan, David Hammond, Malee Fernando, Malcolm Reed, Karen Sisley

**Affiliations:** 1 Department of Oncology, The University of Sheffield, Medical School, Sheffield, United Kingdom; 2 Department of Histopathology, Sheffield Teaching Hospitals, Royal Hallamshire Hospital, Sheffield, United Kingdom; The Institute of Cancer Research, London, United Kingdom

## Abstract

Most soft tissue sarcomas are characterized by genetic instability and frequent genomic copy number aberrations that are not subtype-specific. Oligonucleotide microarray-based Comparative Genomic Hybridisation (array CGH) is an important technique used to map genome-wide copy number aberrations, but the traditional requirement for high-quality DNA typically obtained from fresh tissue has limited its use in sarcomas. Although large archives of Formalin-fixed Paraffin-embedded (FFPE) tumour samples are available for research, the degradative effects of formalin on DNA from these tissues has made labelling and analysis by array CGH technically challenging. The Universal Linkage System (ULS) may be used for a one-step chemical labelling of such degraded DNA. We have optimised the ULS labelling protocol to perform aCGH on archived FFPE leiomyosarcoma tissues using the 180k Agilent platform. Preservation age of samples ranged from a few months to seventeen years and the DNA showed a wide range of degradation (when visualised on agarose gels). Consistently high DNA labelling efficiency and low microarray probe-to-probe variation (as measured by the derivative log ratio spread) was seen. Comparison of paired fresh and FFPE samples from identical tumours showed good correlation of CNAs detected. Furthermore, the ability to macro-dissect FFPE samples permitted the detection of CNAs that were masked in fresh tissue. Aberrations were visually confirmed using Fluorescence in situ Hybridisation. These results suggest that archival FFPE tissue, with its relative abundance and attendant clinical data may be used for effective mapping for genomic copy number aberrations in such rare tumours as leiomyosarcoma and potentially unravel clues to tumour origins, progression and ultimately, targeted treatment.

## Introduction

Leiomyosarcomas belong to a group of sarcomas that are characterised by genetic instability as evidenced by pervasive, seemingly random karyotypic abnormalities. Although the complexity of genomic abnormalities varies significantly among clinically similar cancer cases, certain aberrations have been shown to be recurrent and preserved as the tumours evolve. Accumulating evidence has thus led to the current view that genetic instability is an enabling characteristic that leads to the cancer phenotype and that the resulting recurrent copy number aberrations are an important clue to pathogenetic mechanisms [1], [2]. These recurrent aberrations are believed to result in amplification or deletion of genes that function to promote or inhibit respectively, tumour induction and/or progression. In addition, certain aberrations in remote genomic regions have been shown to frequently occur simultaneously, suggesting that they may not be independent events. Some genes in such regions have been demonstrated to be functionally relevant in major pathways of oncogenesis, e.g. the *ZNF703, DDHD2* and *FGFR1* genes at 8p12 and CCND1 at 11q13 that are amplified in breast tumours [3]. A similar example among sarcomas is the co-amplification of *MDM2, CDK4* and *HMGA2* at 12q14, which have recently been show to occur on separate amplicons in liposarcomas [4].

**Table 1:** A summary of FFPE leiomyosarcoma cases included in this study.

Case	Anatomical Site	Tumour Sampling Date	Age of Sample at Analysis	DLR Spread
*LMS 1*	Bowel	2010	1	0.24
*LMS 2*	Lower Limb	2011	1	0.21
*LMS 3*	Lower Limb	2011	1	0.17
*LMS 4*	Bladder	2011	1	0.17
*LMS 5*	Stomach	2011	1	0.23
*LMS 6*	Stomach	1994	17	0.25
*LMS 7*	Lower Limb	1999	12	0.26
*LMS 8*	Bladder	1998	13	0.21
*LMS 9^§^*	Vagina	2011	1	0.25
*LMS 10^§^*	Retroperitoneum	2011	1	0.29
*LMS 11^§^*	Pelvis	2011	1	0.34
*LMS 12*	Stomach	2011	1	0.26
*LMS 13*	Uterus	2004	8	0.40
*LMS 14*	Bowel	1995	17	0.32
*LMS 15*	Uterus	1997	15	0.35
*LMS 16*	Uterus	1997	15	0.33
*LMS 17*	Nose	1998	14	0.26
*LMS 18*	Pelvis	2004	8	0.21
*LMS 19*	Retroperitoneum	2003	9	0.23
*LMS 20*	Uterus	2008	4	0.21
*LMS 21*	Uterus	2011	1	0.17
*LMS 22*	Lower Limb	2000	12	0.27

DLR Spread – Derivative Log Ratio Spread of Array Data.

§ - Additional fresh samples obtained and frozen before fixing in formalin.

Mapping of genome-wide copy number aberrations can be done by microarray-based comparative genomic hybridisation (array CGH or aCGH). It involves co-hybridising fragments of test and reference genomic DNA that has been differentially labelled with fluorescent dyes to a set of mapped and annotated DNA sequences (probes) on a microarray. By measuring the ratio of fluorescence at each probe, it is possible to detect copy number differences between test (tumour) and reference (normal) DNA at that genomic location. Target probes may be in the form of cDNA sequences, Bacterial Artificial Chromosomes (BACs) or oligonucleotides and depending on the size, type and number of probes on the array, copy number aberrations (CNAs) can be detected at the level of single genes and even specific exons [5]. The highest resolution aCGH methods available are the oligonucleotide (60 mers) arrays and with up to a million probes on an array, CNAs may be detected with a resolution as high as 1–2 Kilobase pairs (Kb). Commercially available oligonucleotide CGH arrays have an added advantage of being easily customisable to focus on specific areas of the genome [6].

In order to generate reproducible aCGH results, pure high molecular weight DNA (usually obtained from fresh frozen tissue, blood or cultured cells) has traditionally been used. Availability of fresh tissue is however limited, particularly in rare tumours like leiomyosarcomas and most tumour tissue available for research is formalin-fixed and paraffin-embedded (FFPE) in order to preserve the tissue structure for histopathology. DNA isolated from such tissues is typically of low quality (low yield and low molecular weight) due to the degradative and fragmenting effects of formalin [7]. Studies comparing the aCGH performance of high and low molecular weight DNA showed that fragment sizes <200 bp (typical of FFPE DNA) produced noisy and irreproducible results [8], [9].

Another major limitation to the use of FFPE DNA for high-resolution oligonucleotide aCGH is technical difficulty in labelling fragmented DNA. Traditional enzymatic methods for labelling DNA (Nick translation or Random priming) involve a fragmentation step with DNase or restriction digestion respectively, which in the case of FFPE DNA further fragments the DNA. An alternative to enzymatic labelling is the Universal Linkage System (ULS), which directly labels the DNA by a chemical reaction that incorporates platinum-conjugated fluorophores into DNA without the need for fragmentation, making it suitable for low molecular weight DNA such as that from FFPE tissue [10].

Recent efforts have been made to optimise the utility of archived FFPE tissues for array CGH after labelling by enzymatic [11] and ULS methods [12], [13], [14], [15], [16]. Nevertheless, the use FFPE DNA for aCGH is still regarded as technically challenging and limited to very small-scale studies, which have reported variable array data quality. In this study, we report the optimisation of the faster, cheaper ULS labelling protocol for high-resolution oligonucleotide array CGH on the analysis of archival FFPE leiomyosarcoma tissue using the Agilent® 180K platform. We were able to compare the quality of the array data with that from DNA obtained from fresh frozen (FF) samples of the same tumours, and used Fluorescence In-situ Hybridisation (FISH) to verify some of the consistent copy number changes.

**Figure 1:**
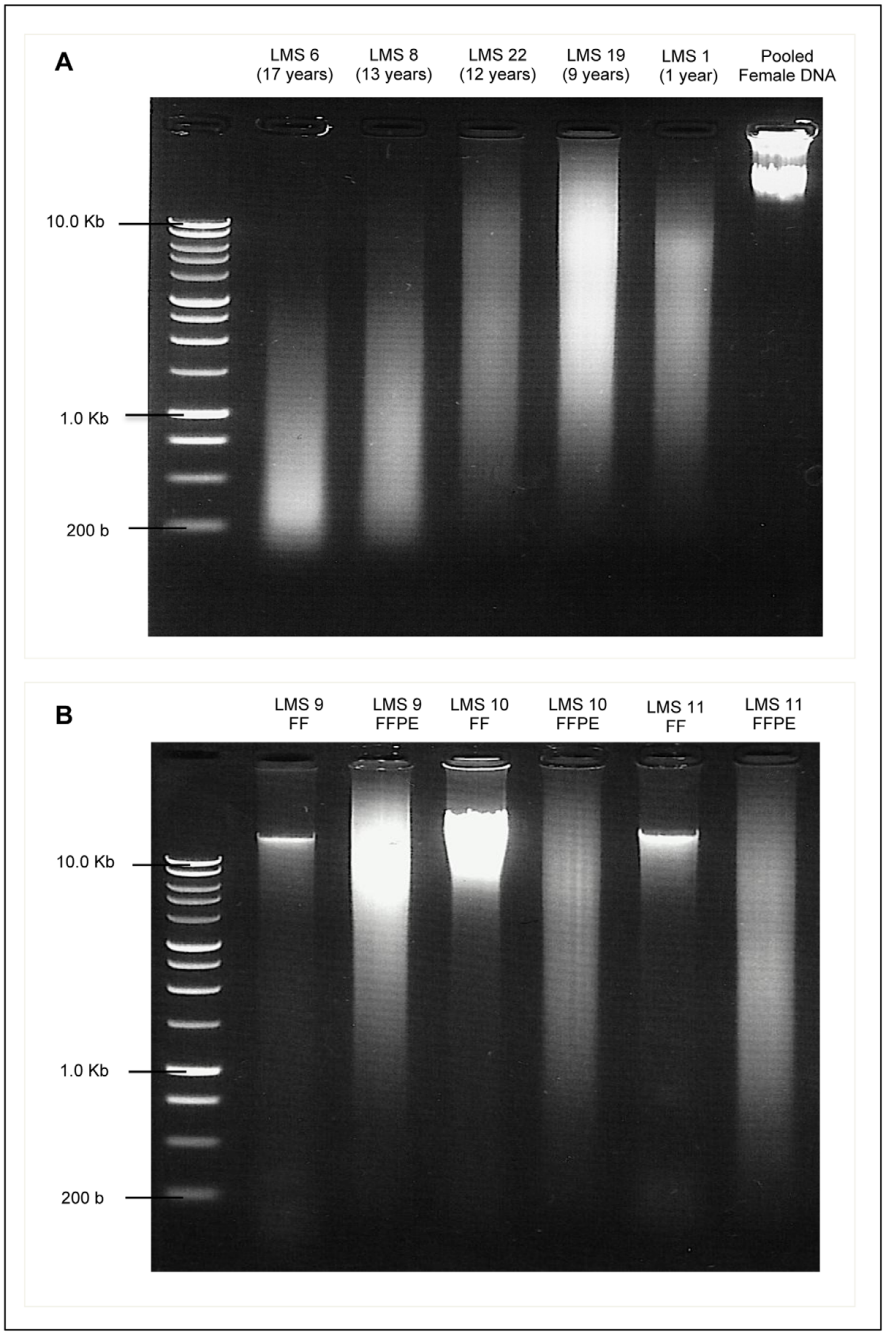
Agarose Gel images of DNA extracted from Leiomyosarcoma Tissue. A: DNA extracted from FFPE leiomyosarcoma samples of different ages (shown in brackets) showing varied degrees of degradation, compared with commercial pooled female genomic DNA. **B:** Comparison of DNA extracted from paired FF and FFPE leiomyosarcoma samples (LMS 9, 10 and 11). FF samples show relatively distinct bands of high molecular weight, while corresponding FFPE samples show low molecular weight fragments in a wide range of sizes. All DNA samples are compared against a 1 Kb DNA ladder. DNA Electrophoresis was done on 1.0% agarose gels were pre-stained with Ethidium Bromide and examined under UV light.

## Materials and Methods

### Ethics Statement

National Research Ethics Committee approval was obtained for the collection and use of fresh and archival tissue samples (reference numbers 09/H1313/52 and 09/H1313/30, respectively). Written informed consent was obtained before the collection of fresh tissue samples and all data from archival samples was analysed anonymously. All tissue was collected and stored according to the principles of the Declaration of Helsinki and the use of tissue was in compliance with the Human Tissue Act 2004.

**Table 2:** Optimisation of DNA Labelling Protocol.

Sample	Tissue Type	Amount of DNA Used (µg)	ULS-Dye Used	DNA to dye ratio (µg/µl)	Post Labelling DNA Yield (µg)	Degree of Labelling (%)	Labelling Pass/Fail	Dye Signal Intensity on Array
***A - Labelling reactions carried out using hot blocks and water baths***								
1	FFPE	0.5	Cy5	**1.0**	0.49	0.4	**Fail**	*NP
2	FFPE	0.5	Cy5	**1.0**	0.5	0.2	**Fail**	*NP
3	FFPE	0.5	Cy5	**1.0**	0.5	0.23	**Fail**	*NP
4	FFPE	0.5	Cy5	**1.0**	0.66	0.36	**Fail**	*NP
5	Control DNA	0.5	Cy3	**1.0**	0.45	0.45	**Fail**	*NP
6	Control DNA	0.5	Cy3	**1.0**	0.45	0.45	**Fail**	*NP
7	Control DNA	0.5	Cy3	**1.0**	0.39	0.39	**Fail**	*NP
8	Control DNA	0.5	Cy3	**1.0**	0.56	0.56	**Fail**	*NP
***B - Labelling reactions carried out using thermal cycler***								
9	FF	0.5	Cy5	**1.0**	0.89	2.01	**Pass**	99
10	FFPE	0.5	Cy5	**1.0**	0.72	2.39	**Pass**	94
11	FFPE	0.5	Cy5	**1.0**	0.59	3.27	**Pass**	79
12	Control DNA	0.5	Cy3	**1.0**	0.73	1.08	**Fail**	*NP
13	Control DNA	0.5	Cy3	**1.0**	0.68	1.15	**Fail**	*NP
14	Control DNA	0.5	Cy3	**1.0**	0.88	0.97	**Fail**	*NP
15	Control DNA	0.5	Cy3	**1.0**	0.83	2.15	**Pass**	153
16	Control DNA	0.5	Cy3	**1.0**	0.78	2.19	**Pass**	148
17	Control DNA	0.5	Cy3	**1.0**	0.82	2.19	**Pass**	103
***C - Labelling reactions carried out using thermal cycler and excess dye***								
18	FFPE	0.8	Cy5	**0.8**	0.8	2.24	**Pass**	307
19	FFPE	0.8	Cy5	**0.8**	0.6	2.32	**Pass**	245
20	FFPE	0.8	Cy5	**0.8**	0.64	1.56	**Pass**	275
21	FFPE	0.8	Cy3	**0.8**	0.91	2.04	**Pass**	383
22	Control DNA	0.8	Cy3	**0.8**	0.95	3.08	**Pass**	1503
23	Control DNA	0.8	Cy3	**0.8**	0.81	3.07	**Pass**	1251
24	Control DNA	0.8	Cy3	**0.8**	0.76	3.1	**Pass**	1228
25	Control DNA	0.8	Cy3	**0.8**	0.87	3.11	**Pass**	1403

* NP – Array CGH **not performed** on sample (if sample failed on spectrophotometry). Spectrophotometry for DNA concentration and Degree of Labelling (DoL) done using Nanodrop® ND-2000 and optimal DoL = 0.75–2.5% (for ULS- Cy5); or 1.75–3.5% (for ULS Cy3). Dye Signal Intensity calculated as part of Quality Control Metrics by Agilent Feature Extraction Software (v10.7.3).

### Tumour Samples and Clinical Data

Formalin-fixed, paraffin-embedded (FFPE) samples of tumour collected between 1997 and 2011 were obtained for 22 leiomyosarcoma cases (Table 1) from the Histopathology Department of the Royal Hallamshire Hospital, Sheffield.

Standard H&E-stained slides representative of tumour blocks were marked to identify areas of >70% viable tumour, which were then macro-dissected by scraping off 20 µm sections on glass slides using a scalpel blade. For three of these cases, prior to fixing in buffered formalin and within 30 minutes of surgical excision, tumour and normal tissue (if available) were macroscopically sampled by an experienced sarcoma pathologist and then snap-frozen. Array CGH experiments were done on paired fresh frozen (FF) and FFPE tumour samples. When normal tissue was not available for sampling, pooled normal genomic DNA (Promega®) was used as reference DNA for array CGH experiments.

### Genomic DNA Extraction

Genomic DNA was extracted from approximately 25 µg of fresh frozen tissue (tumour and normal) using the Qiagen® DNeasy Blood and Tissue Kit, according to manufacturer’s instructions. A modification of the same protocol recommended by the ULS labelling system manufacturer (Agilent®) was used for DNA extraction from FFPE tissues. Briefly, approximately 4 mm^3^ of tissue (the equivalent of two 20 µm-thick sections measuring 10 mm×10 mm) was heat de-paraffinised at 90°C, followed by overnight treatment with 1M-sodium thiocyanate. This was followed by 48-hour proteinase K treatment and then RNase A treatment. DNA was then purified using the Qiagen® DNeasy kit, substituting the wash buffer AW2 with 80% ethanol, and eluting in nuclease–free water.

**Table 3:** Correlation of Probe log_2_ ratios of paired FF and FFPE samples of 3 leiomyosarcoma cases.

*Correlation*	LMS 9	LMS 10	LMS 11
Number of Probes	180,880	180,880	180,880
Pearson Coefficient, r	0.59	0.54	−0.02
95% Confidence Interval	0.5830 to 0.5891	0.5365 to 0.5431	−0.03 to −0.021

Pearson’s Correlation, r of log_2_ ratio values of all probes on tumour DNA samples was calculated using GraphPad Prism software and statistically significant (p<0.001).

### DNA Quantitation and Quality Assessment

Extracted DNA was quantified by spectrophotometry using a Nanodrop ND-1000 (Nanodrop®). Ratios of absorbance, A_260/280_ and A_260/230_ were used to assess DNA purity, and samples with ratios ∼1.80 and >1.90, respectively were regarded as sufficiently pure and suitable for ULS labelling. All DNA samples were visualized on 1.0% agarose gel pre-stained with Ethidium Bromide and fragment sizes were assessed against a 1 Kb DNA ladder (Promega®).

**Figure 2:**
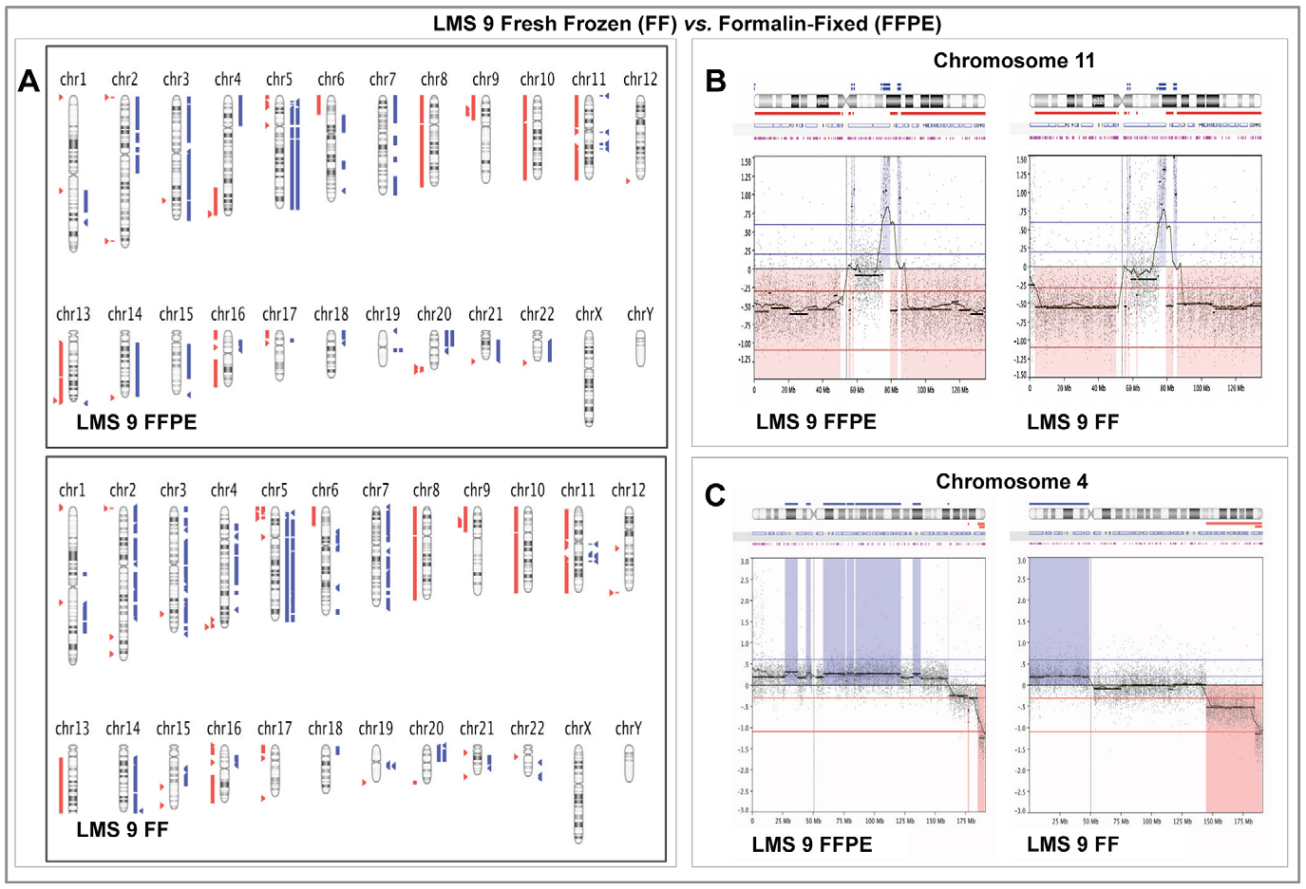
Comparison of Array CGH results in paired Fresh Frozen and Formalin-fixed Paraffin -Embedded samples from LMS 9. **Panel A:** Graphical whole-genome views of copy number aberrations (CNAs) identified in both sample types showing close similarities on most chromosomes. **Panel B:** Higher resolution graphical views of Chromosome 11 showing the close similarity in gain and loss patterns detected in both sample types. **Panel C:** High-resolution views showing the most dissimilar CNA pattern detected between both sample types on chromosome 4. On Panel A, aberrations called by FASST2 algorithm are represented by blue triangles to the right (amplifications) and red triangles to the left (deletions) of the chromosomes. Double blue and red triangles/lines represent high-level amplifications and two-copy deletion, respectively. On Panels B and C, dots represent individual probe log_2_ ratios plotted as a function of their chromosomal position with a moving average of probe log_2_ ratios (wavy dark blue line). Aberration calls are represented by thick black lines with corresponding shaded blue areas above (amplifications) and red areas below (deletions) the zero line.

**Figure 3:**
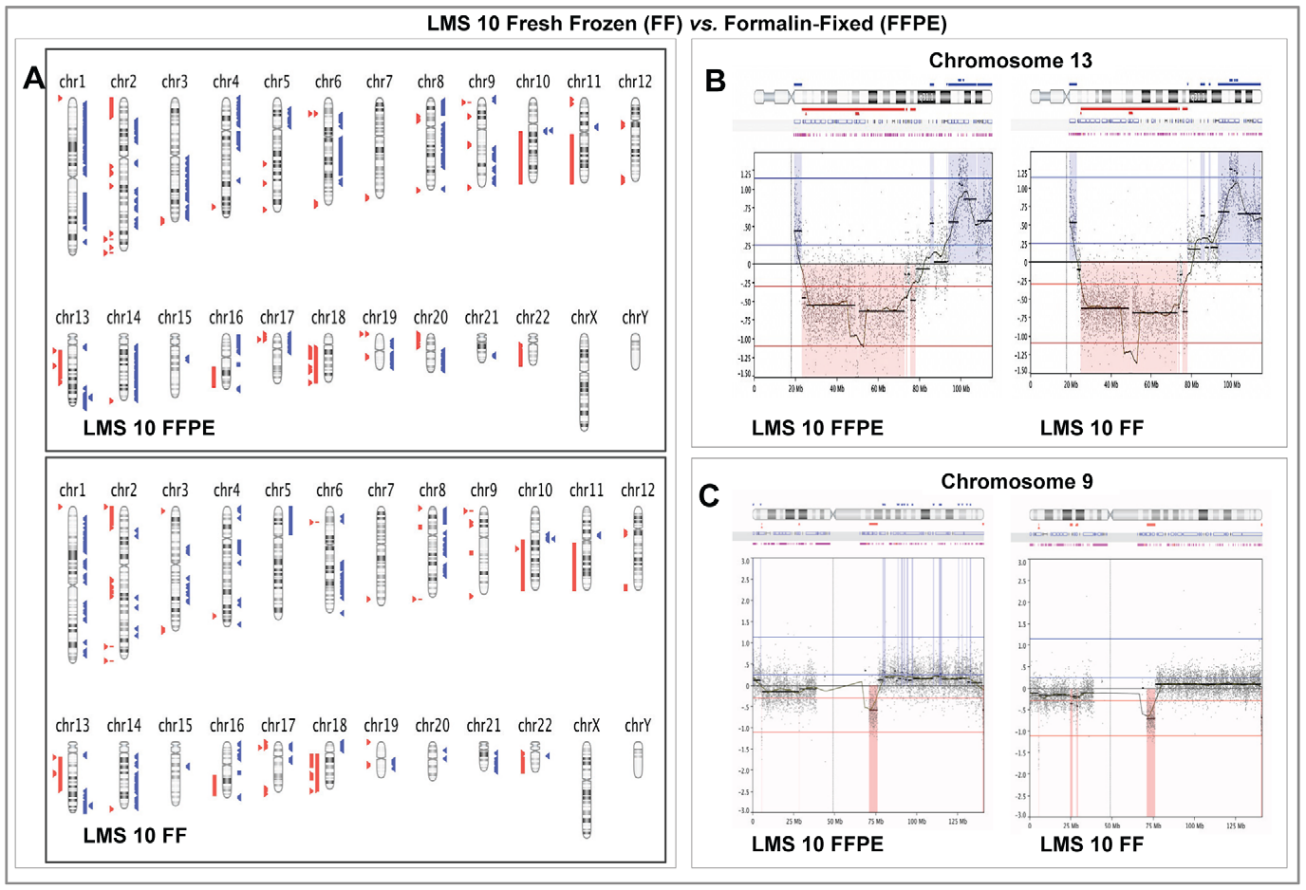
Comparison of Array CGH results in paired Fresh Frozen and Formalin-fixed Paraffin -Embedded samples from LMS 10. **Panel A:** Graphical whole-genome views of copy number aberrations (CNAs) identified in both sample types showing close similarities on most chromosomes. **Panel B:** Higher resolution graphical views of Chromosome 13 showing the close similarity in gain and loss patterns detected in both sample types. **Panel C:** High-resolution views showing that even though the CNAs identified by the calling algorithm on chromosome 9 are not identical, the moving averages of probe log_2_ ratios in both sample types remain similar. On Panel A, aberrations called by FASST2 algorithm are represented by blue triangles to the right (amplifications) and red triangles to the left (deletions) of the chromosomes. Double blue and red triangles/lines represent high-level amplifications and two-copy deletion, respectively. On Panels B and C, dots represent individual probe log_2_ ratios plotted as a function of their chromosomal position with a moving average of probe log_2_ ratios (wavy dark blue line). Aberration calls are represented by thick black lines with corresponding shaded blue areas above (amplifications) and red areas below (deletions) the zero line.

### DNA Labelling

#### Fresh frozen samples

For fresh frozen samples, an enzymatic (random priming) labelling system (Agilent®) was used according to manufacturers’ instructions. Briefly, 0.5–1 µg of genomic DNA was digested with Alu1 and Rsa1 restriction enzymes (Promega®) at 37°C for two hours and then random primers were added. Tumour and normal DNA samples were then labelled with Cy5-dUTP and Cy3-dUTP respectively, by incubation with Exo-Klenow (large fragment of E. coli DNA polymerase I) at 37°C for a further two hours. Excess nucleotides were removed using Amicon 30 µm centrifugal filters (Millipore®).

#### FFPE samples

FFPE tumour and reference DNA was labelled using an optimised version of the protocol for ULS labelling of FFPE DNA (Agilent®). Prior to labelling, heat fragmentation at 95°C was required when average fragment size was greater than 7.0 Kb. 0.5–1 µg of tumour and reference DNA was then chemically labelled by incubating with ULS-Cy5 and Cy3 respectively (about 1 µl dye for 0.8 µg of DNA) in a thirty-minute reaction. Labelling reactions were prepared in thin-walled 0.2 ml PCR tubes and incubated on a thermal cycler with a heated lid (SensoQuest®). Unreacted dye was then removed using KREApure filters (Agilent®).

**Figure 4:**
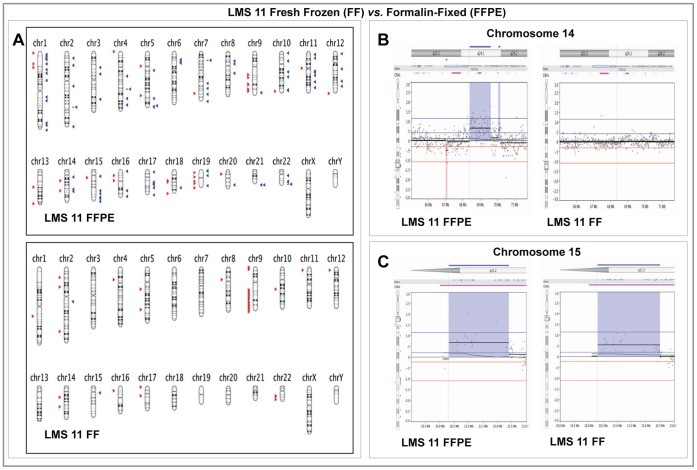
Comparison of Array CGH results in paired Fresh Frozen and Formalin-fixed Paraffin -Embedded samples from LMS 11. **Panel A:** Graphical whole-genome views of both sample types showing that majority of the copy number aberrations (CNAs) identified in the macro-dissected FFPE sample were not detected in the FF sample. Deletions on the long arms of chromosomes 9, 14 and 15 as well as the short arm of chromosome 16 were the called on both sample types. **Panel B:** High resolution graphical views of a 6 Mb region along on Chromosome 14 (14q24.1) showing a group of probes with an average log_2_ ratio of approximately 0.6 and the corresponding single copy amplification detected in the FFPE sample but no aberrations detected in the FF sample. **Panel C:** High-resolution graphical views showing a closely similar copy number aberration detected on Chromosome 15 (15q11.2) in both sample types with similar probe log_2_ ratios. On Panel A, aberrations called by FASST2 algorithm are represented by blue triangles to the right (amplifications) and red triangles to the left (deletions) of the chromosomes. Double blue and red triangles/lines represent high-level amplifications and two-copy deletion, respectively. On Panels B and C, dots represent individual probe log_2_ ratios plotted as a function of their chromosomal position with a moving average of probe log_2_ ratios (wavy dark blue line). Aberration calls are represented by thick black lines with corresponding shaded blue areas above (amplifications) and red areas below (deletions) the zero line.

### Assessment of DNA Labelling Efficiency

Spectrophotometry (Nanodrop ND-2000®) measuring A_260_ (for DNA), A_550_ (for Cy5) and A_649_ (for Cy3) was used for determination of DNA and fluorophore concentrations. The degree of labelling (DoL) is the number of fluorophore molecules per 100 nucleotides, expressed as a percentage and was calculated from the post-labelling DNA yield and fluorophore concentration. According to manufacturer’s recommendations, DoL values between 0.75% and 2.5% were regarded as optimal for Cy5 while values between 1.75% and 3.5% were optimal for Cy3-labelled DNA.

### Array Hybridisation and Scanning

Cy5-Labeled tumour DNA was combined with an equivalent amount of Cy3-labeled reference DNA. In five cases, reference DNA was intentionally sex-mismatched. Repetitive sequences were blocked with human Cot-1 DNA (Invitrogen®) and samples were hybridised onto SurePrint G3 Human CGH Microarrays, 4×180K (Agilent®) according to manufacturer’s instructions. Following hybridisation for 24 hours (FF samples) or 40 hours (FFPE samples), microarray slides were washed according to manufacturer’s instructions and scanned immediately on a DNA Microarray Scanner (Agilent®).

**Figure 5:**
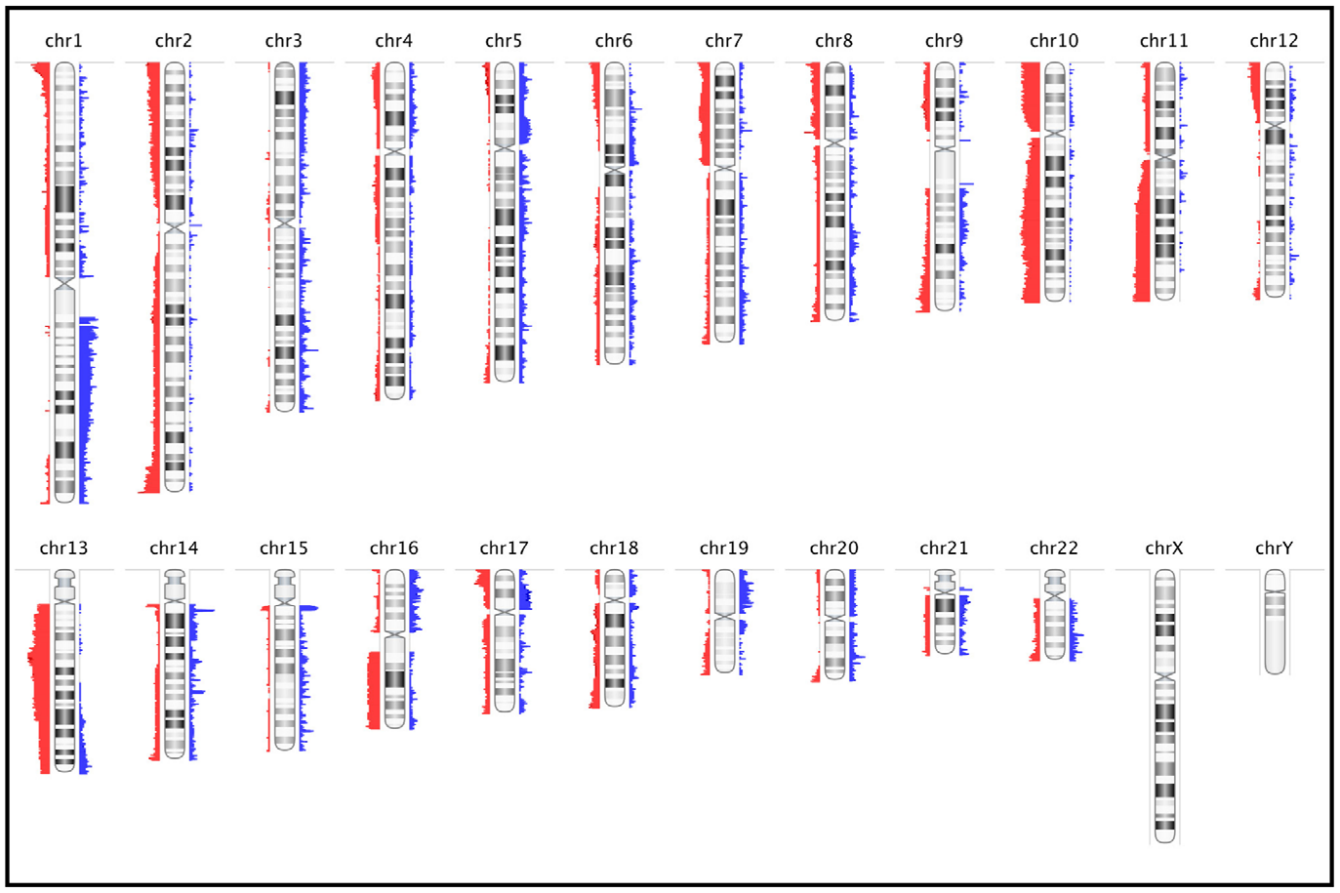
Frequency Plot of Common Genomic Copy Number Aberrations among 22 FFPE Leiomyosarcomas. Commonly aberrant regions are plotted as a function of their chromosomal position. Red bars to the left of the chromosome represent frequency of deletions and blue bars to the right of the chromosome represent amplifications. The heights of the bars correspond to the relative frequency of aberrations among the cases. All CNAs are detected using the FASST2 algorithm.

**Figure 6:**
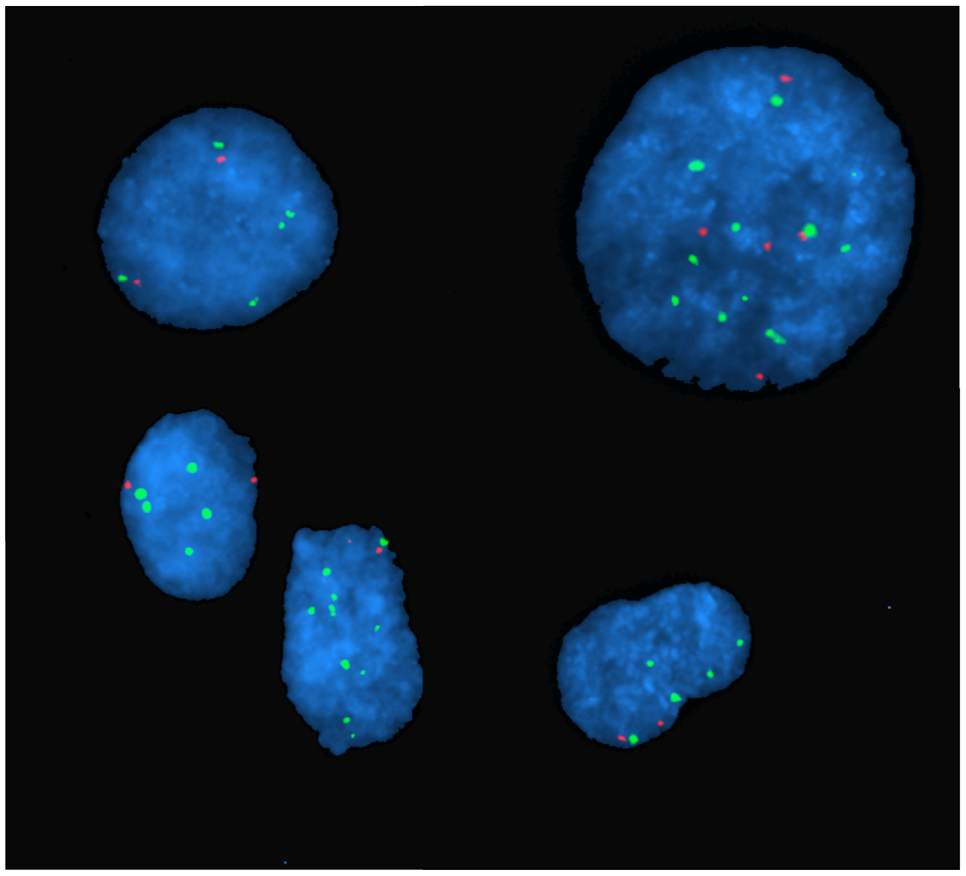
Two-colour Interphase Fluorescence in situ Hybridisation (FISH) Images of nuclei of cultured leiomyosarcoma cells. Most cells have five or more chromosome 11 centromere (green signals), but relatively fewer copies of the ATM region 11q22 (red signals) representing copy number deletion. Nuclei are stained with DAPI (blue). Cells were derived from short-term cultures from fresh tissue (LMS 9).

### Data Analysis

Scanned images were analysed using Feature Extraction software v10.7.3 (Agilent®), which normalizes the fluorescent intensity of both dyes at each probe and calculates their ratio, expressed on a logarithmic scale (probe log_2_ ratio). It also computes a set of Quality Control (QC) metrics including the average green and red signal intensity at all the probes and using non-hybridising control probes, determines the background signal (noise) and signal-to-noise ratio. Average signal intensity >150 with signal-to-noise ratio >20 were regarded as satisfactory.

Among other QC metrics, it calculates the Derivative Log Ratio Spread (DLRS), a measure of the variation in the difference between log_2_ ratios of consecutive probes and other quality control metrics for each array. For paired FF and FFPE DNA samples, Pearson’s correlations of probe log_2_ ratios were calculated using GraphPad Prism Software v5.04 (GraphPad®).

Feature Extracted Data was then analysed using Nexus Copy Number Software v6.1 (Biodiscovery®). For individual arrays, copy number aberrations were called using the FASST2 Segmentation Algorithm, which uses a Hidden Markov Model (HMM)-based approach that does not aim to estimate the copy number state at each probe but uses many states to cover more possibilities, such as mosaic events. These state values are then used to make calls based on a log_2_ ratio threshold. Log_2_ ratio thresholds 0.25 and −0.3 were used to identify single copy number gains and losses respectively, and thresholds for gains and losses of two or more copies were set at 1.14 and −1.1 respectively. Significance threshold p-value for aberration calls was set at a minimum of 5.0×10^−6^, requiring at least three contiguous probes.

For analysis of common aberrations detected among multiple samples, the GISTIC (Genomic Identification of Significant Targets in Cancer) algorithm was used [17]. It was set up to identify areas of the genome with a statistically high frequency of aberration with Q-bound value 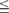 0.05 and G-score cut-off 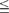
1.0, corrected for multiple testing using False Discovery Rate (FDR) correction [18]. CNA calls on sex chromosomes were excluded from analysis.

### Fluorescence *in situ* Hybridisation (FISH)

Two-colour interphase FISH was used to confirm array CGH results in one of the LMS cases (LMS 9). FISH experiments were carried out on short-term cultured tumour cells using the LSI ATM probe (Vysis®; SpectrumOrange) and an α-satellite probe to the centromere of chromosome 11 (Vysis®, SpectrumGreen), as previously described [19].

**Figure 7:**
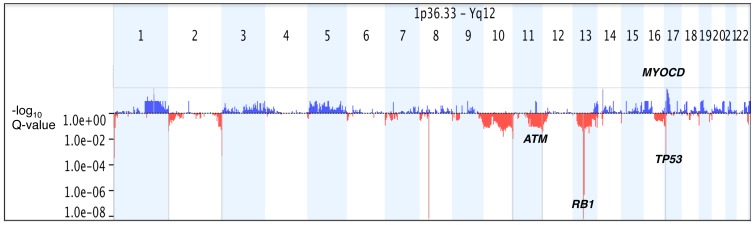
Statistically-Significant Common Genomic Copy Number Aberrations among 22 FFPE Leiomyosarcomas. Statistically significance of common aberrations was determined using the GISTIC algorithm. Commonly aberrant regions are plotted along the x-axis as a function of their chromosomal position and their q-values are plotted on the y-axis on a negative log_10_ scale so that the highest bars represent most significant genomic regions. Blue bars represent commonly amplified regions and red bars represent commonly deleted regions. Genomic regions with G-score >10 and q-values <0.05 are considered significant (shaded grey) and important candidate genes in these regions e.g. RB1, MYOCD are shown in black. Aberrations in individual samples were called using FASST2 Algorithm.

## Results

### DNA Quality

The DNA yield in most FFPE LMS tumours was good, exceeding 10 µg in most cases. In two cases however, the yield was low and vacuum centrifugation was used to bring DNA concentration to optimal values. Another FFPE DNA sample had A_260/230_<1.50 and the DNA was re-purified by sodium acetate-isopropanol precipitation before labelling.

When visualized on agarose gels against a 1 Kb ladder, DNA from the FF samples and commercial pooled genomic DNA showed relatively distinct bands of high molecular weight DNA, while that from FFPE tumours showed a range of fragment sizes that varied from <1.0 Kb on average to as high as 8.0 Kb. The degree of fragmentation appeared to be worse in FFPE samples that were older when compared with the more recent tumours (Figure 1). Tumour samples presented in this study were chosen to reflect a wide range of both sample age and degree to DNA degradation as visualised on agarose gels.

### Optimisation of DNA Labelling

Initial attempts at DNA labelling by the ULS method produced a variable degree of labelling. We noted that when labelling reactions were carried out on heat blocks or water baths, the degree of labelling was consistently low and in cases where post-labelling DNA quantitation showed that there was a higher amount of DNA in the reaction than initially estimated, the degree of labelling was either variable or arrays failed due to signal intensities that were below recommended thresholds. Since the ULS system does not amplify the DNA, this suggested that the ratio of DNA to ULS Cy-dye in the reaction was high, resulting in an inefficient labelling reaction. We therefore modified the protocol to use excess ULS Cy-dye relative to amount of DNA (about 0.8 µg of DNA to 1 ml of dye) and carried out reactions in thin-walled tubes on a thermal cycler to ensure uniform optimal temperature for the reactions. All FFPE samples that were labelled in this way had consistently good degree of labelling. Quality assessment of arrays using this DNA showed good red and green signal intensity that was similar to that seen with high quality DNA that was labelled using the enzymatic method. Results of experiments carried out before and after optimisation of the labelling protocol are summarised in Table 2.

### Array Quality

Signal intensities of both red and green dyes were much higher than the manufacturer-recommended threshold for all DNA samples regardless of source tissue or labelling method. The Derivative Log Ratio Spread (DLRS) is widely regarded as a robust parameter for measurement of the quality of microarray experiments. It represents the ‘noisiness’ of array data and a low DLRS means that the data has small probe-to-probe variability and better ability to array to identify small aberrations and vice versa. Manufacturer-recommended thresholds for DLRS when using FFPE DNA is <0.4, a value above which array data may be compromised and the array should be failed. DLRS values for all the arrays fell in the recommended pass range and all but five of the 22 FFPE DNA arrays had DLRS <0.3, the threshold recommended for DNA from fresh frozen tissue or cells (Table 1).

### Comparison of Array CGH on Fresh Frozen vs. FFPE Tumour DNA

In three LMS cases (LMS 9, 10 and 11), paired samples of macroscopically sampled fresh tumour and macro-dissected FFPE tumour were obtained. High molecular weight DNA from the fresh frozen (FF) tissue was labelled by the enzymatic method, while fragmented FFPE DNA from the same tumours was labelled using the one-step ULS method. There was good correlation between paired FF and FFPE samples in two out of the three cases. The Pearson correlation coefficients (r) of overall probe log_2_ ratios were 0.58 (p<0.0001) and 0.54 (p<0.0001) for LMS 9 and LMS 10 respectively (Table 3).

Genomic profiles of detected CNAs in both sample types were also were also very similar in both LMS cases with most chromosomes showing near identical loss and gain patterns (Figures 2 and 3). One of the most significant differences in CNAs was seen in LMS 9, where a low level amplification detected on chromosome 4 in the macro-dissected FFPE sample was not seen in FF sample (Figure 2C). Similar moving average patterns of higher amplitude were however retained at the telomeric ends of 4q in both sample types. In both LMS 9 and 10, on most chromosomes where the aberrations detected by the calling algorithm were dissimilar, closer examination showed that the moving average pattern of probe log_2_ ratios remained similar with amplitude close to the threshold set in the algorithm for CNA detection. An example is shown in Figure 3C.

The third leiomyosarcoma case (LMS 11) compared in this way however, showed poor overall probe log_2_ ratio correlation (r = −0.02). Although a few small aberrations were seen in both sample types, most of the CNAs detected in the macro-dissected FFPE tissue were not detected in FF tissue (Figure 4). Histological examination showed that unlike the other two cases, the LMS 11 sample was composed of less than 50% tumour cells with a significant ad-mixture of normal cells.

### Common Aberrations

Most of the tumours showed complex genomic profiles, with CNAs detected on ten or more chromosomes. Only three cases had relatively simple genomic profiles. In general, copy number losses were more common than gains across the entire genome. The most frequent aberrations (seen in >40% of cases) include whole or near-whole arm deletion in 10p, 10q, 13q, 16q, and deletion of the telomeric end of 11q (Figure 5). Deletion in 13q14.2– q14.3 was one of the most frequent focal aberrations detected and was present in 14 out of 22 (64%) cases. The most frequent amplification was on 1q21– q23. A number of focal aberrations correlated very closely with known non-pathological copy number variations (CNV) or involved non-coding genomic regions. The frequent deletion in the 11q22 region covering the locus of the ATM gene was confirmed using Fluorescence *in situ* Hybridisation (FISH) in two LMS cases, one on FFPE tissue (not shown) and the other in cultured cells (Figure 6).

Statistical significance of common aberrations was determined using the GISTIC algorithm. A G-score is assigned to each aberrant genomic region based on the frequency and magnitude (log_2_ ratio values) of aberrations. It also determines the statistical probability that the common aberrations occur by chance alone and using False Detection Rate correction, determines a q-value. The program also identifies within these regions, ‘peak’ regions that have the highest statistical likelihood of containing affected genes (maximal G-score and minimal q-value). Using G-score and q-value thresholds of 1.0 and 0.05 respectively, we identified five significant regions of copy number gain covering 331 genes (4 genes in peak region) and seven significant regions of copy number loss covering 452 genes (13 in peak region), shown in Figure 7.

## Discussion

High-resolution mapping of copy number aberrations in cancer genomes is a valuable way of identifying recurrent genomic changes. Coupled with epigenetic, expression and functional data, this can further our understanding of the molecular basis of cancers and markers can be identified that can be targeted for tumour diagnosis, prognostic sub-classification or pharmacologic/biologic therapy [2]. To this end, large-scale projects to catalogue genomic copy number aberrations in large cohorts of specific cancers e.g. the Cancer Genome Atlas Project have been established and over time have yielded important insights in a number of cancers, including glioblastoma multiforme and ovarian carcinoma [20], [21].

Traditionally, such large-scale projects have been designed as prospective studies and exclusively utilise fresh, frozen tumour tissue because of the need for high quality DNA for copy number analysis. Prospective study design is a problem with rare cancers such as LMS because it would take many years to accumulate large enough numbers. Tissue fixation in formalin is the standard procedure in most institutions, and over many years, large FFPE tissue archives have been accumulated. Such archives are an essential source of tumour tissue for research and they come complete with associated clinical data such as disease progression and therapeutic response that can readily be correlated with molecular genetic data.

Formalin fixation, which is aimed primarily at preserving tissue protein structure for histopathological studies results in the formation inter- and intra-strand cross-links between DNA molecules that results in low yields of highly fragmented nucleic acids [7]. Other effects such as strand cleavage and base modifications make PCR amplification of whole genome DNA prone to bias and errors. As expected, DNA from the FFPE LMS tumours in this study showed varying degrees of fragmentation compared to that from FF tissues. The degree of degradation appeared to be worse with older samples. However, factors such as pre-fixation and intra-fixation durations and tissue penetration are known to influence the degree of formalin effects on tissue DNA [7] and some of the more degraded samples were fixed before standardised protocols for tissue fixation to preserve their suitability for molecular studies were widely-established.

Our initial attempts to label FFPE DNA using standard ULS protocols were not consistently successful. We found that the use of a thermal cycler with a heated lid gave higher degrees of labelling than heat blocks or circulating water baths for incubations during labelling, presumably because the temperature is more uniformly maintained throughout the labelling reaction. Insufficient dye amounts relative to DNA were also found to lead to variable or poor ULS labelling, and even small errors (from user or equipment) that caused underestimation of DNA concentration gave poor labelling results. Using an excess of dye relative to DNA in labelling reactions gave consistent good degree of labelling and successful arrays. This is in keeping with results published in a recent study that showed that estimation of DNA concentration is critical to sample assessment for labelling [22].

Regardless of sample age, all aCGH experiments (FF and FFPE) in this study showed good DLRS values when labelled using the modified protocols. In addition, FFPE samples from female patients that were hybridised against sex-mismatched DNA showed the X-chromosome gain or loss with log_2_ ratios near the expected values, even in cases where there were few other genomic aberrations. A number of recent studies using FFPE tissues of similar age have reported variable DLRS values [11], [15], [16], [23]. To our knowledge however, such consistent good DLRS values have not been reported from ULS-labelled FFPE DNA.

Two out of three paired FF and FFPE samples from identical tumours that were compared showed good overall probe log_2_ ratio correlation with Pearson’s coefficients similar to those reported from a similar study [22]. In these two cases, the CNAs detected across the entire genome in the compared FF and FFPE samples were also similar. For a few chromosomes where the CNAs detected by the calling algorithm were dissimilar, visual examination at a higher resolution showed that in most cases a similar moving average pattern was retained and the amplitude of average probe log_2_ ratios was close to the set thresholds for low-level aberration detection, thus explaining why aberrations were differentially called in the two sample types. The calling algorithm thresholds set for analysis of array CGH data in this study were chosen based on previous studies in literature and regarded as valid as they enabled the detection of common aberrations among the LMS cases that concur with previous studies (see below).

The third case (LMS 11) showed poor statistical correlation of the overall probe log_2_ ratios from both sample types and the results showed that although a few common CNAs were seen, the majority of CNAs detected in the FFPE sample were not detected in the FF one. A minority of the genomic regions in LMS 9 and 10 also showed significant difference in the probe moving average pattern in addition to CNA detected. This prompted a revisit of the histology of all three tumours, which showed that LMS 11 was very heterogenous and contained large areas of haematoma and normal cells, while the former two were composed relatively homogenously of tumour cells.

In any whole genome nucleic acid isolation, the presence of germ-line DNA from normal cells ‘contaminating’ a tumour sample can potentially mask genomic aberrations. Our ability to macro-dissect tumour cells from the FFPE tissues apparently helped to reduce the masking of genomic copy number aberrations. Heterogeneity of tumour cell populations between the two areas of the whole tumour that were sampled independently and represented by the FF and FFPE samples could potentially account for some of the low level differential aberrations detected in these paired samples in the cases that looked more homogenous on histological examination.

We carried out common aberration analysis among the 22 FFPE LMS cases in this study. Frequent common aberrations detected were in concordance with those reported in previous studies. Deletions on 10q, 13q and 16q have been reported as frequent among LMS in numerous studies [24], [25], [26], [27]. We have previously demonstrated a frequent loss on 11q that involves the locus of the ATM gene that is mirrored in the current study [19] and confirmed by FISH. Using the GISTIC method, we identified focal genomic regions with a statistically high frequency of copy number aberrations over the “background” aberration. Our results are very similar to those reported by Barretina et al, who showed deletions on 10p, 10q, 13q, 17p and an amplification on 17p as the most statistically significant common aberrations from data obtained from fresh frozen tumour samples [28]. The regions identified contain loci for well-established tumour suppressor and cell cycle regulatory genes such as PTEN, RB1 and TP53. We also identified a focal amplification on 17p that specifically covered most of the MYOCD gene locus that was recently shown to be frequently amplified and over-expressed in at least one subset of leiomyosarcomas [29].

Over a quarter of the cases presented in this study showed CNAs on genomic regions that involve loci for at least three of the five genes mentioned above, and may well represent a subset of leiomyosarcoma, although the small number of cases does not allow any correlation with clinical data to have statistical significance. The potential for expanding such a retrospective study to improve its statistical power cannot however, be overemphasized.

At present, the cost per sample of labelling DNA by the ULS method is less than that of the enzymatic method. In addition, DNA labelling and clean-up is complete within one hour, compared with the enzymatic methods that require at least five times that duration. Most importantly however, in a 24-month period that has seen only three operable leiomyosarcoma cases treated in our centre with the possibility of obtaining fresh tissue, we have been able to access the FFPE archives and analyse more than twenty cases for which progression and survival data is available.

In summary, we have optimised a reliable method that is cheap, fast and gives us access to long-term archival samples prepared using even non-standard protocols. We have been able to generate results from this archival tissue that are in close concordance with those from multiple previous studies that utilised fresh tumour tissue. We therefore now have the option to select specific LMS and other tumour subtypes including those that are very rare for high-resolution genomic copy number mapping.
